# Cholera Infantum

**Published:** 1875-05

**Authors:** E. H. Gibbs


### CHOLERA INFANTUM

Is the most fatal of all diseases that prevail in this country. During the months of June, July, August and September, its alarming fatality among children under three years old, is a reproach to the medical profession. It baffles the skill of every school of practice. All meet on the same level here, and all seem powerless before the march of this dreaded disease. Untold thousands of little children, each season, find relief from their sufferings only in death.

For the sake of little children, who occupy the largest space in every true human heart; for the sake of the bereaved, who are represented in almost every family in the land, and whose constant fear is that they may be compelled to make farther sacrifices to this relentless "moloch;" for the credit of the medical profession, and of civilization itself, it is time some check were put upon the ravages of this fearful disease. In ordinary affairs, "to be forewarned, is to be forearmed." But here is an exception to the rule. We know as well on the first day of each new year as on the last, how many little graves will be required for the victims of "CHOLERA INFANTUM." The disease is far more fatal in cities than in country places, and in those localities where little regard is paid to sanitary rules. But it is an error to suppose that the disease does not prevail in country villages remote from the great centres of population.

The disease is so well known, and its existence so easily recognized, even by laymen, that a description of it would be superfluous, were it not to serve as a warning to its approach, which is always insidious.

When a child is seized with vomiting and purging attended with great suffering, the parents usually insist that it was taken suddenly ill, and that it was perfectly well a few hours before. In this they are almost always mistaken. The disease has its period of incubation like all other diseases, and it is important to recognize its existence at this period, in order to cure it, and save the little patient from unnecessary suffering.

Whatever the primary cause, whether the irritation of the gums from teething, or confinement in hot and illy-ventilated rooms, the actual seat of the disease is in the LIVER; and the active treatment should be almost exclusively directed to that organ. The moment this fact is recognised, the disease loses its terrors.

Mistakes in diagnosis have made mourners in all lands from the time of Galen to the present, but it is doubtful whether any simple disease was ever so little understood and so unskillfully treated as "Cholera Infantum" or "Summer Complaint of Children." Physicians have the highest medical authority for treating the symptoms of the disease instead of the disease itself; and what is generally recognised as the regular treatment is far more hazardous than leaving the disease to nature and to chance, and this is the reason. The opium, in some form, which is always given to allay pain and check the vomiting and purging, still farther paralyzes the liver. Its healthy action is suspended; the opium checks the vomiting and purging and pain; the stomach and bowels, and frequently the brain and lungs, become gorged with fluids and the child *unexpectedly* dies just after its symptoms have been pronounced more favorable.

The history of this disease is but a repetition of the history of many other diseases that were terribly fatal until physicians learned how to treat them.

It is but a few years since the report that "asiatic cholera" was on its march westward, produced the greatest alarm, and paralyzed the business of the country. Small-pox was a terror to the nations. Incipient disease of the lungs was interpreted as a "death warrant." Simple "spinal irritation" was regarded as the premonition of a lingering death. These diseases have been stripped of their terrors since they have become well understood and the proper remedies applied.

Thus "Cholera Infantum," the saddest of them all, because the most widely disseminated and the most fatal, and because also of the utter helplessness of the little sufferers, will lose its terrors when the causes and nature of the disease and its proper treatment come to be *generally* understood.

Finally, it should be generally understood that opium in every form—even under the insidious and deceitful name of "Soothing Syrup"—should never be given to a child under fifteen years of age for any purpose, *except to kill him.* To give it in "Cholera Infantum" is criminal. It necessarily aggravates the disease, and the death of the patient can oftener be attributed to its use than to the disease; and its recovery is far more likely, by an entire neglect of treatment, than by any treatment in which opium in any form is employed.

The best course to pursue when a child first presents decided symptoms of "Summer Complaint," is to dress it in flannel underclothing and take it into the country, where it can get fresh air and fresh, wholesome food. It will generally recover in two or three days, often without medicine. The symptoms are almost certain to return if taken home again too soon. Comparatively few children, however, can be taken into the country. Those that are compelled to remain are the ones that demand our solicitude and attention.

Preliminary to all other treatment, the surroundings must be made clean and comfortable. Flannel underclothing, particularly over the stomach and bowels, is essential. Wholesome and nutritious food should be provided and given, a little at a time, as the judgment of the physicians or nurse may dictate. The treatment must be directed to the liver. As soon as that is stimulated to normal action, the danger is passed, and convalescence certain and rapid.

To meet cases of "Cholera Infantum," and diarrhœa of children, Messrs. Hall & Ruckel of this city have put up very neatly, in the form of sugar pellets, a prescription, called "Van Deren's Remedy for Cholera Infantum and Summer Complaint" of children. It can be found at Druggists throughout the country. It claims not to be a patent medicine, but simply the prescription of an experienced physician. I have employed the "Remedy" with the most satisfactory results, and cheerfully add my testimony to that of many other physicians who have thoroughly tested it, and recommend it as a perfectly reliable remedy.

E. H. Gibbs, A.M., M.D.

---

Growth of Man.—Observations regarding the rate of growth of man have determined the following interesting facts: The most rapid growth takes place immediately after birth, the growth of an infant during the first year of its existence being about eight inches. The ratio of increase gradually decreases until the age of three years, at which time the size attained is half that which it is to become when full grown. After five years the succeeding increase is very regular till the sixteenth year, being at the rate for the average man of two inces a year. Beyond sixteen the growth is feeble, being for the following two years about six-tenths of an inch a year; while from eighteen to twenty the increase in height is seldom over one inch. At the age of twenty-five the growth ceases, save in a few exceptional cases. It has furthermore been observed that, in the same race, the mean size is a little larger in cities than in the country, a fact that will be received with doubt by many who have come to regard the rustic as the true model man.

